# Sites of metastasis and association with clinical outcome in advanced stage cancer patients treated with immunotherapy

**DOI:** 10.1186/s12885-019-6073-7

**Published:** 2019-08-29

**Authors:** Mehmet Asim Bilen, Julie M. Shabto, Dylan J. Martini, Yuan Liu, Colleen Lewis, Hannah Collins, Mehmet Akce, Haydn Kissick, Bradley C. Carthon, Walid L. Shaib, Olatunji B. Alese, Conor E. Steuer, Christina Wu, David H. Lawson, Ragini Kudchadkar, Viraj A. Master, Bassel El-Rayes, Suresh S. Ramalingam, Taofeek K. Owonikoko, R. Donald Harvey

**Affiliations:** 1 Department of Hematology and Medical Oncology, Emory University School of Medicine, Atlanta, GA USA; 2 Department of Hematology and Medical Oncology, Winship Cancer Institute of Emory University, 1365 Clifton Rd, Atlanta, GA USA; 3 Departments of Biostatistics and Bioinformatics, Emory University, 1518 Clifton Rd, Atlanta, GA USA; 4 Department of Urology, Emory University, 5673 Peachtree, Dunwoody Rd, Atlanta, GA USA; 5 Department of Pharmacology, Emory University School of Medicine, 1365 Clifton Rd, Atlanta, GA USA

**Keywords:** Immunotherapy, Phase 1 clinical trials, Sites of metastasis, Liver metastasis, Clinical outcomes, Tumor immunology, Tumor microenvironment, Immune checkpoint blockade

## Abstract

**Background:**

Selecting the appropriate patients to receive immunotherapy (IO) remains a challenge due to the lack of optimal biomarkers. The presence of liver metastases has been implicated as a poor prognostic factor in patients with metastatic cancer. We investigated the association between sites of metastatic disease and clinical outcomes in patients receiving IO.

**Methods:**

We conducted a retrospective review of 90 patients treated on IO-based phase 1 clinical trials at Winship Cancer Institute of Emory University between 2009 and 2017. Overall survival (OS) and progression-free survival (PFS) were measured from the first dose of IO to date of death or hospice referral and clinical or radiographic progression, respectively. Clinical benefit (CB) was defined as a best response of complete response (CR), partial response (PR), or stable disease (SD). Univariate analysis (UVA) and Multivariate analysis (MVA) were carried out using Cox proportional hazard model or logistic regression model. Covariates included age, whether IO is indicated for the patient’s histology, ECOG performance status, Royal Marsden Hospital (RMH) risk group, number of metastatic sites, and histology.

**Results:**

The median age was 63 years and 53% of patients were men. The most common histologies were melanoma (33%) and gastrointestinal cancers (22%). Most patients (73.3%) had more than one site of distant metastasis. Sites of metastasis collected were lymph node (*n* = *58*), liver (*n* = *40*), lung (*n* = *37*), bone (*n* = *24*), and brain (*n* = *8*). Most patients (80.7%) were RMH good risk. Most patients (*n = 62*) had received 2+ prior lines of systemic treatment before receiving IO on trial; 27 patients (30.0%) received prior ICB. Liver metastases were associated with significantly shorter OS (HR: 0.38, CI: 0.17–0.84, *p* = 0.017). Patients with liver metastasis also trended towards having shorter PFS (HR: 0.70, CI: 0.41–1.19, *p* = 0.188). The median OS was substantially longer for patients without liver metastases (21.9 vs. 8.1 months, *p* = 0.0048).

**Conclusions:**

Liver metastases may be a poor prognostic factor in patients receiving IO on phase 1 clinical trials. The presence of liver metastases may warrant consideration in updated prognostic models if these findings are validated in a larger prospective cohort.

## Background

The emergence of immunotherapy (IO) has transformed the clinical landscape for the treatment of patients with advanced cancers of various histologies [1–5]. As of July 2018, the US Food and Drug Administration (FDA) has approved six immune checkpoint blockers (ICB) for advanced cancer patients. These agents target CTLA-4 (ipilimumab), PD-1 (nivolumab, pembrolizumab), or PD-L1 (atezolizumab, avelumab, and durvalumab) and are used as monotherapy as well as in combination with other anti-cancer drugs [2, 6–8]. These agents have a more favorable toxicity profile than chemotherapy or targeted therapies and offer the promise of durable clinical benefit, albeit only for a minority of patients [9–13].

As the list of IO options continues to expand [14], selecting the appropriate patients to receive IO represents a critical area of research. Biomarkers of response previously explored include angiopoietin-2 (ANGPT2) in melanoma and polybromo-1 (PBRM1) and polybromo-associated barrier-to-autointegration factor (PBAF) in renal cell carcinoma (RCC) [6, 15]. In lung cancer, bladder cancer, and RCC, PD-L1 expression has been associated with response to ICB [16–19]. Additionally, in lung cancer, tumor mutational burden has been investigated as a potential biomarker for responsiveness to IO-based therapies [20, 21]. In breast cancer, levels of tumor-infiltrating lymphocytes may be prognostic [22, 23]. The identification of a uniform prognostic and predictive biomarker of response to IO across various cancer types remains an unmet need in oncology.

Royal Marsden Hospital (RMH) risk scoring, which incorporates albumin < 3.5 g/dL, lactate dehydrogenase > the upper limit of normal, and > two sites of metastasis, has been shown to accurately predict survival in patients treated on phase 1 clinical trials across various cancer types [24–26]. While the RMH scoring system predicts that the number of metastatic sites affects clinical outcomes, investigation into differential prognosis between specific metastatic sites in IO therapy is lacking.

Previous studies have established that prognosis for patients with liver metastasis is poor in those with primary colorectal, bladder, and breast cancer [27–31]. Based on the literature that liver metastases point to a worse prognosis in various cancers, we hypothesized that the specific sites of metastatic disease may affect survival in patients enrolled onto IO-based phase 1 clinical trials. In this study, we investigated the association between sites of metastatic disease of various primary histologies and clinical outcomes in patients enrolled on IO-based phase 1 clinical trials.

## Methods

We retrospectively reviewed the electronic medical records of 90 patients with advanced cancer treated on IO-based phase 1 clinical trials between 2009 and 2017 at the Winship Cancer Institute of Emory University. Data collected from electronic medical records included: demographic information, medication allergies, Eastern Cooperative Oncology Group (ECOG) performance status (PS), histology, number and site of distant metastases, number and type of prior lines of systemic therapy, prior treatment with ICB, best response to IO on trial, date of radiographic or clinical progression, immune-related adverse events, date of death or last follow-up, and RMH risk factors. Response to treatment was determined by using Response Evaluation Criteria in Solid Tumor version 1.1 by centralized review. The sites of distant metastases that were collected from review of clinic notes and baseline radiology reports included brain, lung, liver, lymph node, and bone.

This data review and analysis was approved by the Emory University Institutional Review Board (IRB), and waiver of consent was granted due to the retrospective nature of this study. All patients provided written informed consent for the phase 1 clinical trial to which they were enrolled, which were also reviewed and approved by the Emory University IRB.

### Statistical analysis

Clinical outcomes were measured using three variables: overall survival (OS), progression-free survival (PFS), and clinical benefit (CB). OS and PFS were measured from the first dose of IO to date of death and clinical or radiographic progression, respectively. For patients who were referred to hospice but did not have confirmed dates of death, date of hospice referral was used in place of date of death. In this cohort, 54 patients had confirmed dates of death, while 9 patients had a documented date of hospice referral without a confirmed date of death. Clinical benefit (CB) was defined as a best response of complete response (CR), partial response (PR), or stable disease (SD) for at least one restaging scan. Median duration of SD for patients in this cohort was 6.7 weeks, with a range of 3.3 to 70.6 weeks. Progressive disease (PD) was defined as a patient coming off trial for declining performance status due to clinical progression.

Statistical analysis was conducted using SAS Version 9.4 and SAS macros developed by the Biostatistics and Bioinformatics Shared Resource at Winship Cancer Institute [32]. The significance level was set at *p* < 0.05. The univariate association (UVA) with different sites of metastasis of each covariate used the chi-square test or Fisher’s exact for categorical covariates and ANOVA for numerical covariates. The Multivariate analysis (MVA) of OS or PFS was tested by proportional hazard model, with hazard ratio (HR) and its 95% confidence interval (CI) being reported. The multivariable model was built by controlling for age, gender, allergies, race, the patient’s primary histology, ECOG PS, RMH risk group, history of diabetes, prior IO, number of prior therapies, and number of distant metastatic sites following by a backward selection procedure with a removal criterial of alpha > 0.05. Similar strategy was used to fit logistic regression model for CB.

## Results

Patient demographic information and disease characteristics are presented in Table 1. The majority of patients (58.9%) in this retrospective cohort of 90 patients were men. The most common histology was melanoma (33.3%), followed by gastrointestinal (GI) cancers (22.2%), and lung and head & neck cancers (20.0%). More than half of the patients (*n* = *46*, 51.1%) received an FDA-approved ICB combined with an experimental IO agent, 27.8% (*n* = *25*) of patients received anti-PD-L1 monotherapy, and 21.1% (*n = 19*) received an experimental IO agent as monotherapy. Most patients (*n = 62*, 68.9%) had received two or more prior lines of systemic treatment before receiving IO on trial; 27 patients (30.0%) received prior ICB. The majority of patients (80.7%) were RMH good risk while 17 patients were RMH poor risk at the start of IO.

**Table 1:** Baseline Characteristics and Demographics of Patients

	*n* (%)
Gender	
Male	53 (58.9)
Female	37 (41.1)
Race	
White	70 (77.8)
Black	16 (17.8)
Asian/Unknown	4 (4.4)
Histology	
Melanoma	30 (33.3)
Gastrointestinal	20 (22.2)
Lung, Head & Neck	18 (20.0)
Breast	11 (12.2)
Gynecological cancers	3 (3.3)
Genitourinary cancers	3 (3.3)
Others	5 (5.6)
Number of metastatic sites	
1	24 (26.7)
2	33 (36.7)
3+	33 (36.7)
Sites of metastases	
Lymph node	58 (64.4)
Liver	40 (44.4)
Lung	37 (41.1)
Bone	24 (26.7)
Brain	8 (8.9)
ECOG PS	
0	34 (38.2)
1	55 (61.8)
RMH Risk Group	
Good	71 (80.7)
Poor	17 (19.3)
Checkpoint Indication	
Yes	49 (54.4)
No	41 (45.6)
Treatment Regimen	
Anti-PD-L1 Monotherapy	25 (27.8)
FDA-approved IO + Experimental IO	46 (51.1)
Experimental IO Monotherapy	19 (21.1)
Number of prior systemic therapies in the metastatic setting	
0–1	28 (31.1)
2+	62 (68.9)
Prior treatment with ICB	
Yes	27 (30.0)
No	63 (70.0)

*ECOG PS* Eastern Cooperative Oncology Group performance status, *RMH* Royal Marsden Hospital, *IO* Immunotherapy, *PD-L1* Programmed death ligand 1, *ICB* Immune checkpoint blocker

Most patients (73.3%) had more than one site of distant metastasis. Sites of metastasis recorded were lymph nodes (*n = 58*), liver (*n = 40*), lung (*n = 37*), bone (*n = 24*) and brain (*n = 8*). Metastasis to each of these sites was analyzed for association with OS, PFS, and CB.

**Fig. 1:**
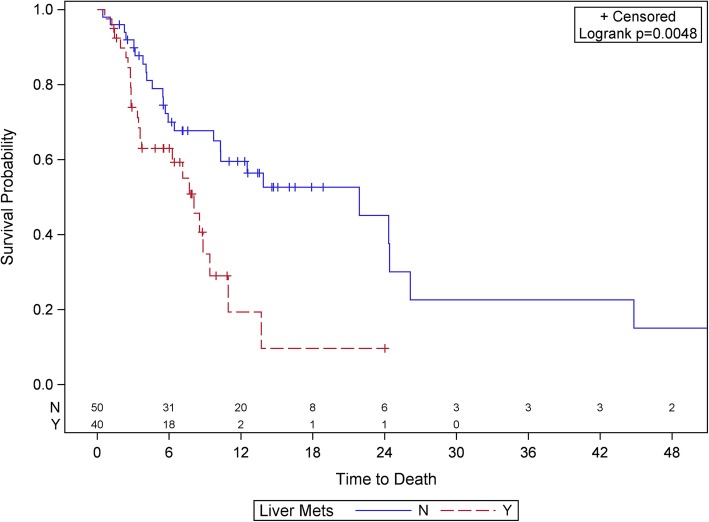
Kaplan-Meier plot of overall survival (OS) stratified by presence of liver metastases

**Fig. 2:**
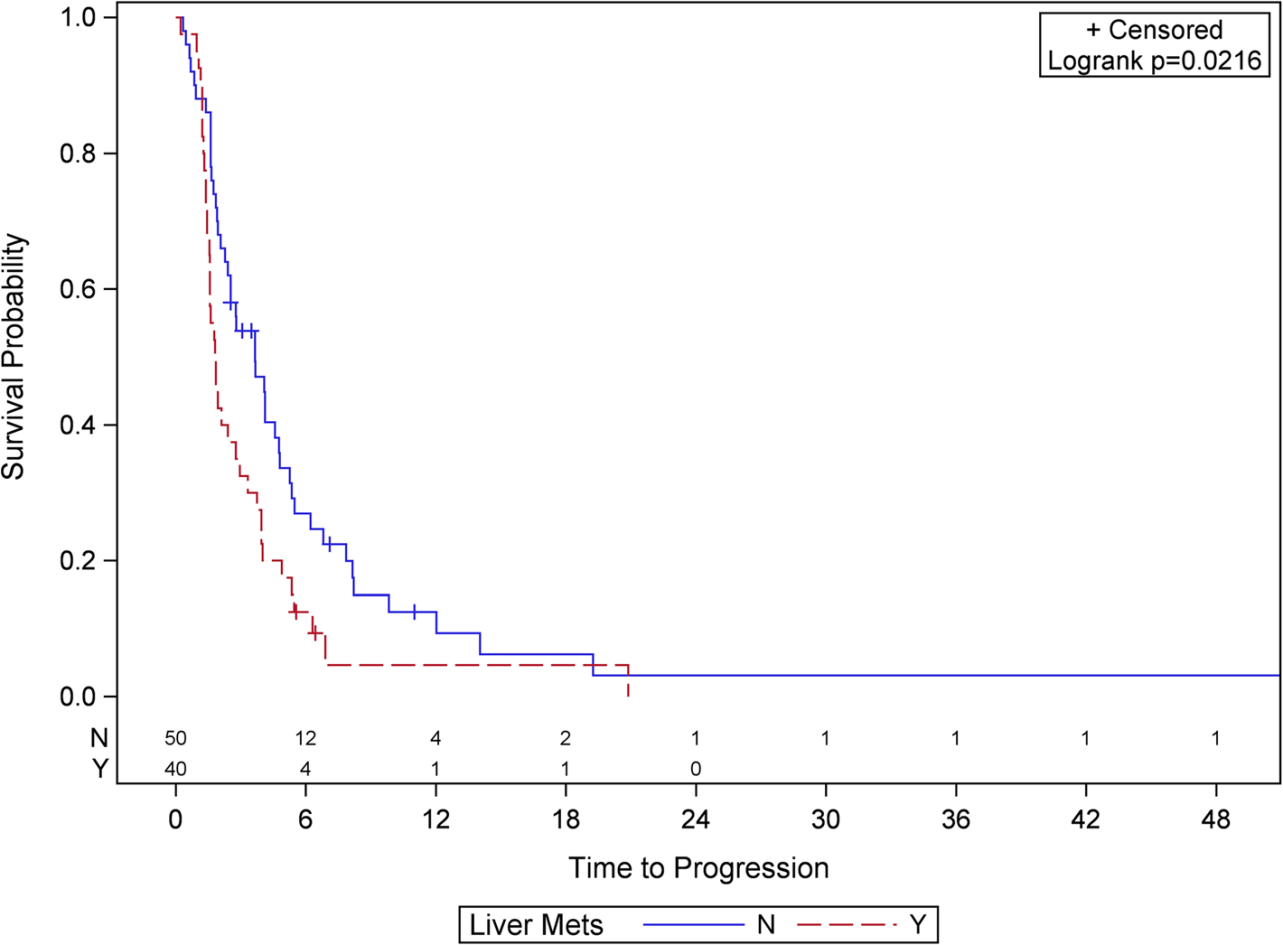
Kaplan-Meier plot of progression-free survival (PFS) stratified by presence of liver metastases

UVA of total number of and sites of metastatic disease with clinical outcome are provided in Tables 2 and 3**,** respectively. The presence of liver metastasis was significantly associated with shorter OS, PFS, and lower rate of CB in UVA (all *p* < 0.03). Other sites of metastatic disease were not significant in UVA. Therefore, we built an MVA using liver metastases as a risk factor, provided in Table 4. In MVA, patients with liver metastases had significantly shorter OS (HR: 0.38, CI: 0.17–0.84, *p* = 0.017) and trended towards having shorter PFS (HR: 0.70, CI: 0.41–1.19, *p* = 0.188), regardless of patients’ primary histologies. The median OS was substantially longer for patients without liver metastases (21.9 vs. 8.1 months, *p* = 0.0048). The Kaplan-Meier plot of the association between liver metastases and OS and PFS are shown in Fig. 1 and Fig. 2**,** respectively.

**Table 2:** UVA of number of metastases with clinical outcome

	OS		PFS		CB	
Number of Metastases	HR (CI)	*p*-value	HR (CI)	*p*-value	OR (CI)	*p*-value
1 (*n* = 24)	0.47 (0.22–1.01)	0.054	0.60 (0.35–1.05)	0.072	4.37 (1.40–13.64)	0.011*
2 (*n* = 33)	0.39 (0.20–0.78)	0.007*	0.45 (0.27–0.77)	0.003*	4.24 (1.48–12.17)	0.007*
3+ (*n* = 33)	–	–	–	–	–	**–**

*UVA* Univariate analysis, *OS* overall survival, *PFS* progression-free survival, *CB* clinical benefit, *HR* Hazard Ratio, *CI* Confidence Interval, *OR* Odds Ratio

*statistical significance at alpha < 0.05

**Table 3:** UVA of sites of metastases with clinical outcome

Site of Metastasis	OS		PFS		CB	
	HR (CI)	*p*-value	HR (CI)	*p*-value	OR (CI)	*p*-value
No lymph node metastases (*n* = 32)	1.42 (0.79–2.54)	0.244	1.16 (0.74–1.83)	0.524	0.73 (0.31–1.76)	0.486
Lymph node metastases (n = 58)	–	–	–	–	–	–
No bone metastases (*n* = 66)	0.61 (0.32–1.17)	0.135	0.80 (0.48–1.32)	0.376	2.00 (0.75–5.31)	0.164
Bone metastases (*n* = 24)	–	–	–	–	–	–
No liver metastases (*n* = 50)	0.42 (0.23–0.78)	0.006*	0.60 (0.39–0.93)	0.024*	2.64 (1.11–6.28)	0.028*
Liver metastases (*n* = 40)	–	**–**	–	**–**	–	**–**
No brain metastases (*n* = 82)	0.69 (0.29–1.64)	0.406	0.86 (0.40–1.88)	0.712	1.44 (0.32–6.42)	0.633
Brain metastases	–	–	–	–	–	–
No lung (*n* = 53)	1.02 (0.57–1.82)	0.944	1.20 (0.76–1.87)	0.433	1.17 (0.50–2.73)	0.713
Lung metastases (*n* = 37)	–	–	–	–	–	–

*UVA* Univariate analysis, *OS* overall survival, *PFS* progression-free survival, *CB* clinical benefit, *HR* Hazard Ratio, *CI* Confidence Interval, *OR* Odds Ratio

*statistical significance at alpha < 0.05

**Table 4:** MVA† of liver metastases with clinical outcome

	OS		PFS		CB	
	HR (CI)	*p*-value	HR (CI)	*p*-value	OR (CI)	*p*-value
No liver metastases (*n* = 50)	0.38 (0.17–0.84)	0.017*	0.70 (0.41–1.19)	0.188	1.42 (0.39–5.21)	0.597
	Median: 21.9 months 12 month survival: 60%		Median: 3.6 months 12 month survival: 13%		Rate: 56% (0 CR, 6 PR, 22 SD, 17 PD, 5 NE)	0.026*
Liver metastases (*n* = 40)	Median: 8.1 months 12 month survival: 19%		Median: 1.8 months 12 month survival: 5%		Rate: 33% (1 CR, 1 PR, 11 SD, 24 PD, 3 NE)	–

*MVA* Multivariate analysis, *OS* overall survival, *PFS* progression-free survival, *CB* clinical benefit

† Covariates considered in MVA initially include age, gender, ECOG PS, prior IO, number of prior therapies, RMH risk group, race, number of metastatic sites and primary histology. Backward selection procedure was implemented by removal criterial of *p* > 0.05. The final controlled variables are primary histology and RMH risk group for OS and PFS and primary histology, race, and number of prior therapies for CB. *MVA* Multivariate analysis, *OS* overall survival, *PFS* progression-free survival, *CB* clinical benefit, *HR* Hazard Ratio, *CI* Confidence Interval, *OR* Odds Ratio

*statistical significance at alpha < 0.05 by Chi-square test

Patients with reported liver metastasis most commonly had primary GI tumors (47.5%); non-GI tumors included melanoma (27.5%), lung and head & neck (10%), breast (7.5%), and gynecologic (2.5%). Patients without reported liver metastasis most commonly had primary melanoma (38%) and lung and head & neck tumors (28%). Of the patients with liver metastases, 71.8% were RMH good risk at the start of IO. Most patients with liver metastases (72.5%) had received two or more lines of systemic therapy prior to treatment with IO. Patients with metastatic disease in the liver were more likely to have a greater total number of sites of metastatic disease. One half (50%) of the patients with liver metastases had a total of three or more distant metastases while only 26% of patients without liver metastases had three or more distant metastatic sites.

## Discussion

In this study, we demonstrated that metastasis to the liver is associated with worse clinical outcomes in advanced stage cancer patients treated on IO-based phase 1 clinical trials. Regardless of tumor histology, patients in this cohort with documented metastasis to the liver had shorter OS and PFS and a lower rate of CB. The results from this study build upon previous studies that have explored the predictive value of metastatic sites in cancers treated with chemotherapy, particularly in breast, bladder, and colon cancer [27–31, 33]. In this study we assessed different sites of metastatic disease and clinical outcomes in patients treated with IO-based regimens as part of phase 1 clinical trials, which has not been investigated previously. The results support the Pires da Silva et al. study findings that in melanoma patients who receive combination immunotherapy, different metastatic sites exhibit different effects on survival, and patients with liver metastases experience inferior clinical responses [34]. Our cohort of patients receiving IO-based therapy in phase 1 clinical trials is a unique population. The cohort includes patients with several different primary cancer types rather than just one. Furthermore, patients enrolled onto phase 1 clinical trials receive novel IO agents, which is another reason to investigate this cohort of patients.

Evidence suggests that primary tumor histology influences prognosis for patients with metastasis to the liver who are treated with chemotherapy. Jaffe et al. (1968) found that primary tumor site influences prognosis for patients with hepatic metastases [35]. Furthermore, Soni et al. (2015) found that subtypes of breast cancer differ in their metastatic behavior [36]. The results of our study, however, suggest that for patients on IO-based phase 1 clinical trials, regardless of primary tumor site, liver metastases are a poor prognostic indicator. This may be explained biologically by the liver’s immuno-regulatory behavior [37]. The liver, notably located between the genitourinary circulation and systemic circulation, functions as a secondary lymphoid organ. It contains a high density of natural killer T-cells as well as T-regulatory cells [37, 38]. Therefore, metastases to the liver may interfere with the immune-regulatory behavior of the organ, which in turn affects the response of cancer patients on IO. The mechanism by which this occurs should be explored further.

The presence of metastatic disease in the liver has been established as a poor predictive factor for patients receiving chemotherapy-based treatment and has thus merited different or more aggressive treatment for patients with liver metastases. Previous studies have found that patients with breast and colorectal cancer with metastases to the liver may receive clinical benefit from liver resection [39–43]. Given these previous findings in cohorts treated with chemotherapy, patients with solitary liver metastases may benefit from liver resection prior to starting IO. However, many patients in our study cohort with advanced stage cancers of various primary tumor histologies had multiple liver metastases, making liver resection not clinically appropriate. Priestman and Hanham (1972) found that combination chemotherapy produces longer overall survival rates than single chemotherapy in treating patients with breast or colorectal cancer with liver metastases [44]. Using these results in chemotherapy-based treatment as a model, clinical outcomes for patients on IO-based therapy may improve with combination chemotherapy or targeted therapy to the liver prior to or in addition to IO. Additionally, radiation therapy to the liver prior to initiating IO could improve clinical outcomes in patients with hepatic metastases, as per the abscopal effect [45–47].

Our analysis has limitations to note. This is a retrospective study, which is inherently subject to selection bias. We attempted to mitigate this bias by including all patients who received at least one dose of IO on a phase 1 clinical trial at our institution. Due to our lenient inclusion criteria, the patient population was very heterogeneous in primary tumor histology and in type of IO received. We accounted for this by controlling for primary tumor histology and other baseline disease characteristics. Though the size of our patient cohort may limit the impact of this study, given our lenient inclusion criteria, the study cohort was the largest cohort of patients receiving immunotherapy as part of phase 1 clinical trials at our institution. Additionally, only the five most common sites of metastasis were captured and analyzed independently. We did not differentiate between isolated metastases to the liver versus widespread metastatic disease. There were very few patients with brain metastases, so the predictive value of brain metastases could not be adequately analyzed. Finally, patients enrolled onto phase 1 clinical trials likely have further advanced disease than patients who receive immunotherapy in the first or second line, which limits the generalizability of this study.

## Conclusions

Liver metastases are a poor predictive factor in this cohort of patients treated on IO-based phase 1 clinical trials. Patients in the retrospective cohort with hepatic metastases had shorter OS, PFS and lower rate of CB. If these findings are validated in a larger study, this baseline disease characteristic may warrant consideration in updated prognostic models for stratification of patients enrolled onto IO-based phase 1 clinical trials. The presence of liver metastases should not preclude patients from enrolling onto phase 1 trials. Rather, the results of this study reveal an important area for improvement in IO-based therapies for advanced stage cancer patients with hepatic metastases. Further advancements in treating these patients are needed. The detection of liver metastasis in advanced stage cancer patients may be especially useful in determining whether these patients should receive novel combination therapy or should receive liver-targeted therapy prior to or in combination with IO, given the unique microenvironment around metastatic tumors in the liver.

## Data Availability

The datasets used and/or analyzed during the current study are available from the corresponding author on reasonable request.

## Abbreviations

CB: Clinical benefit
CI: Confidence interval
CR: Complete response
CTLA-4: Cytotoxic T-lymphocyte associated protein 4
ECOG: Eastern Cooperative Oncology Group
FDA: Food and Drug Administration
GI: Gastrointestinal
HR: Hazard ratio
ICB: Immune checkpoint blocker
IO: Immunotherapy
MVA: Multivariate analysis
OS: Overall survival
PD-1: Programmed cell death protein 1
PD-L1: Programmed death ligand 1
PFS: Progression-free survival
PR: Partial response
PS: Performance status
RCC: Renal cell carcinoma
RMH: Royal Marsden Hospital
SD: Stable disease
UVA: Univariate analysis
